# Supplementary material for: AlcoR: alignment-free simulation, mapping, and visualization of low-complexity regions in biological data
Source: Gigascience. 2023 Dec 13;12:giad101. doi: 10.1093/gigascience/giad101 (PMC10716826; doi:10.1093/gigascience/giad101)
Supplement: giad101_Supplemental_File [file giad101_supplemental_file.pdf]

Supplementary material of
"AlcoR: alignment-free simulation, mapping and visualization of low-complexity regions in biological data"

J. M. Silva, W. Qi, A. J. Pinho and D. Pratas

1 Supplementary Tables and Figures

	Local		Local and Distant		
Virus	Proportion (%)	Total	Proportion (%)	Total	Length
HSV-1	2.1	3280	20.9	31838	152222
HSV-2	2.1	3359	20.7	32048	154675
VZV	0.8	1119	12.4	15587	124884
EBV	19.0	32901	20.2	34936	172764
HCMV	0.2	540	3.0	7089	235646
HHV6A	3.9	6346	13.4	21434	159378
HHV6B	3.6	5966	13.4	21801	162114
HHV7	7.6	11785	14.3	21979	153080
KSHV	2.5	3464	3.6	5017	137969

Table S1: Viral genomes low-complexity quantities. Local stands for a $c = 5000$.

	Local		Local and Distant		
Chromosome	Proportion (%)	Total	Proportion (%)	Total	Length
1	8.3	20803967	15.8	39270479	248387328
2	1.5	3878233	6.6	16047585	242696752
3	2.8	5692943	7.9	16046400	201105948
4	3.3	6440097	8.5	16612930	193574945
5	2.7	4950774	8.9	16355103	182045439
6	1.9	3424293	6.8	11810528	172126628
7	3.5	5643328	11.4	18423589	160567428
8	1.8	2768397	7.7	11393460	146259331
9	20.8	31428854	30.5	45980087	150617247
10	3.1	4271161	8.2	11067525	134758134
11	3.3	4560817	9.1	12313590	135127769
12	2.6	3596703	7.4	9975181	133324548
13	10.0	11395149	16.8	19166013	113566686
14	7.0	7122783	15.6	15880032	101161492
15	13.5	13518221	24.8	24790491	99753195
16	16.2	15627718	26.6	25647061	96330374
17	6.2	5254793	14.9	12619600	84276897
18	6.9	5637641	9.8	7897841	80542538
19	10.0	6218589	24.7	15274375	61707364
20	7.8	5166656	11.8	7819509	66210255
21	11.0	4994285	23.0	10393899	45090682
22	20.4	10516642	35.2	18105816	51324926
23	2.7	4258258	14.3	22062353	154259566
24	59.0	36860460	76.5	47801487	62460029

Table S2: Human chromosomal low-complexity quantities. Local stands for a $c = 5000$.

	A		B		
Chromosome	L	L+D	L	L+D	Length
1	0.6	37.4	0.5	36.1	44602016
2	0.4	36.7	0.8	39.9	39759535
3	0.5	32.3	0.2	32.3	34098055
4	2.5	39.6	1.4	39.3	35070039
5	0.7	34.0	0.7	32.9	33994110
6	1.1	37.4	0.9	37.7	32064973
7	5.3	45.6	7.8	45.7	37888301
8	1.6	44.6	1.8	46.5	40936035
9	0.9	43.6	0.8	39.0	39491830
10	2.2	41.0	1.4	38.0	33530900
11	0.4	34.2	0.4	33.7	34309032
12	4.7	45.2	1.7	42.0	40282042
13	1.3	47.9	0.9	46.2	39960896
14	1.2	35.7	0.7	33.6	31289863
15	1.8	37.8	2.7	38.0	35497395
16	0.4	40.6	0.3	40.7	34021704
17	5.2	53.0	2.7	48.4	37533413
18	11.6	52.1	1.3	47.4	37650304

Table S3: Cassava chromosomal low-complexity percentages for both haplotypes (A and B). "L+D" stands for local and distant low-complexity while "L" stands for a local low-complexity with $c = 5000$.

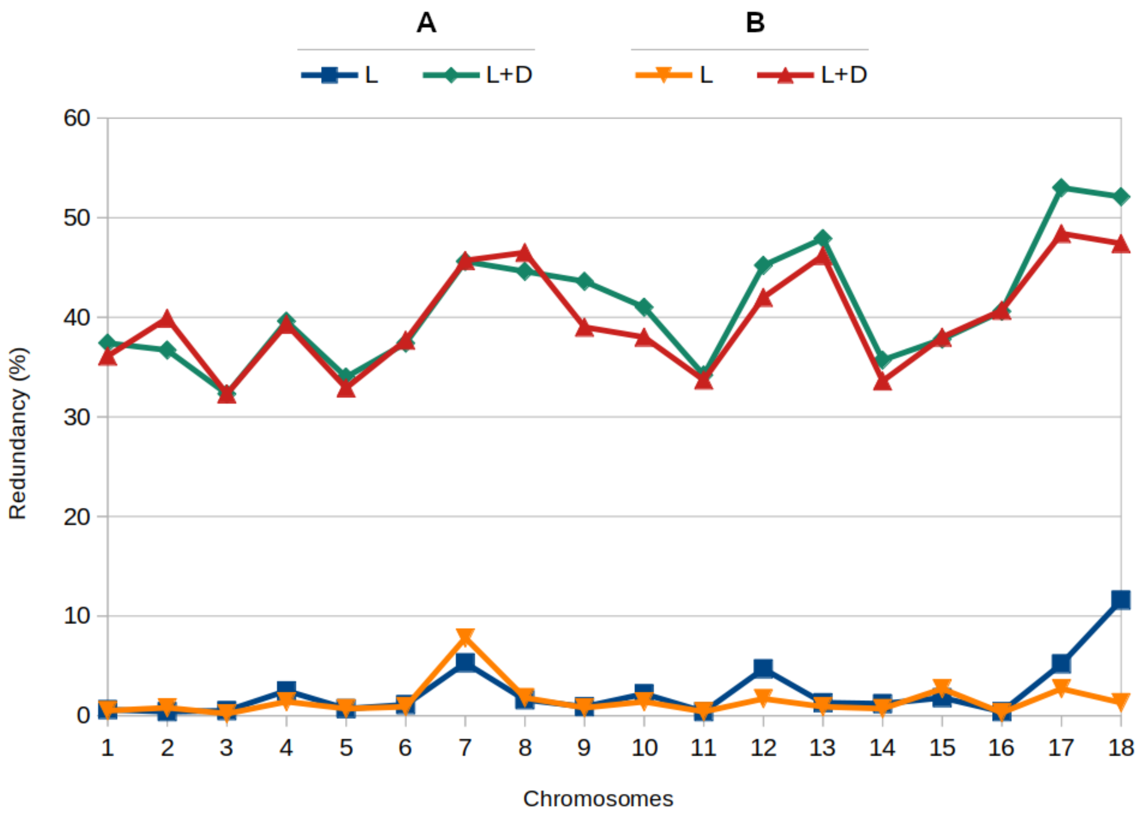

Figure S1: Cassava low-complexity proportion in percentage for both haplotypes (A and B). "L+D" stands for local and distant low-complexity while "L" stands for a local low-complexity with $c = 5000$.

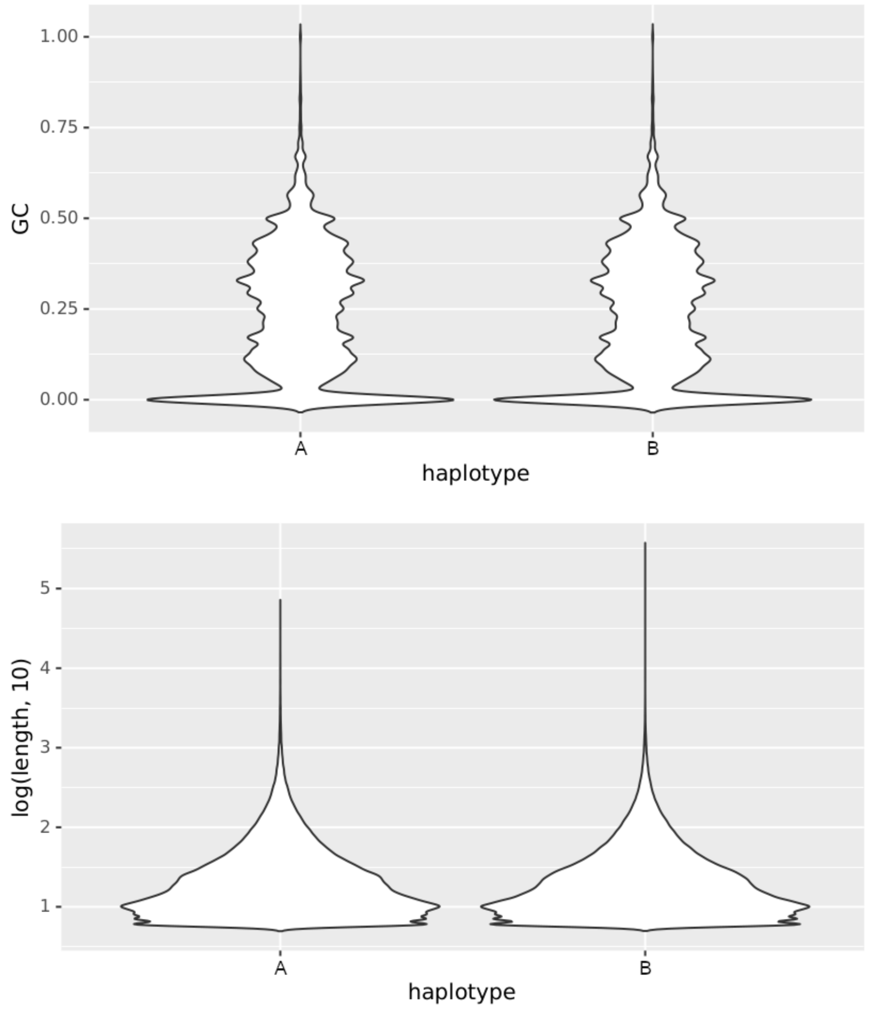

Figure S2: Cassava low-complexity regions according to GC and length for A and B.

2 Reproducibility

2.1 Installation

To install AlcoR using the bioconda [1], the following command must run

```
conda install -c bioconda alcor -y
```

Alternatively, this procedure can be performed using the code available at the repository as

```
git clone https://github.com/cobilab/alcor.git
cd alcor/src/
cmake .
make
```

Please, use the alternative way to reproduce the experiments described at the article and below.

2.2 Reproducing the "Demonstration A"

These instructions assume that the code has been compiled with the alternative way under the src/ folder. Then, in the demos/ folder of the AlcoR repository, please, type

```
cd demoA/
cp ../../src/AlcoR .
chmod +x *.sh
./GenSeqA.sh
./DemoA.sh
```

The output is provided in the same folder, including the outA.pdf file with the results image.

Specifically, the synthetic sequence contains the sub-sequences in the following order

- **R1** - 1k length pseudo-random sequence (seed: 13);
- **R2** - region A, 2k length pseudo-random sequence (seed: 1);
- **R3** - 2k length pseudo-random sequence (seed: 1401);
- **R4** - region A inverted, I(A);
- **R5** - 2k length pseudo-random sequence (seed: 27);
- **R6** - region A with 5% of substitution mutations, M(A, 5%);
- **R7** - 2k length pseudo-random sequence (seed: 17);
- **R8** - region A with 10% of substitution mutations, M(A, 10%);
- **R9** - 2k length pseudo-random sequence (seed: 311);
- **R10** - region A, a complete 2k copy;
- **R11** - 1k length pseudo-random sequence (seed: 713).

2.3 Reproducing the "Demonstration B"

These instructions assume that the code has been compiled with the alternative way under the src/ folder. Then, in the demos/ folder of the AlcoR repository, please, type

```
cd demoB/
cp ../../src/AlcoR .
chmod +x *.sh
./GenSeqB.sh
./DemoB.sh
```

The output is provided in the same folder, including the outB.pdf file with the results image.

Specifically, the synthetic sequence contains the sub-sequences in the following order:

- **R1** - 2k length HHV6B sequence from 32k to 34k and inverted (seed: 3);
- **R2** - 2k length generated sequence with an FCM of context order 7 and a bet of 100 and trained from B19V (seed: 1);
- **R3** - 2k length HHV6B sequence from 5k to 7k and inverted (seed: 7);
- **R4** - 2k length generated sequence with an FCM of context order 7 and a bet of 100 and trained from B19V (seed: 1), followed by a substitution mutational of 1% (seed: 1);
- **R5** - 2k length HHV6B sequence from 42k to 44k (seed: 11);
- **R6** - 2k length generated sequence with an FCM of context order 7 and a bet of 100 and trained from B19V (seed: 31), followed by a substitution mutation of 1% (seed: 31);
- **R7** - 2k length HHV6B sequence from 11k to 13k and inverted (seed: 13);
- **R8** - 2k length generated sequence with an FCM of context order 7 and a bet of 100 and trained from B19V (seed: 71);
- **R9** - 2k length pseudo-random sequence (seed: 83);
- **R10** - 2k length B19V sequence from 2k to 4k (seed: 91);
- **R11** - 2k length pseudo-random sequence (seed: 97);
- **R12** - 2k length B19V sequence from 2k to 4k and inverted (seed: 91), followed by a deletion mutation of 15% (seed: 91);
- **R13** - 2k length pseudo-random sequence (seed: 101);
- **R14** - 2k length B19V sequence from 2k to 4k and inverted (seed: 91), followed by a addition mutation of 10% (seed: 1);
- **R15** - 2k length pseudo-random sequence (seed: 103);

2.4 Reproducing the “Demonstration C”

These instructions assume that the code has been compiled with the alternative way under the src/ folder. Then, in the demos/ folder of the AlcoR repository, please, type

```
cd demoC/
cp ../../src/AlcoR .
chmod +x *.sh
./GenSeqC.sh
./DemoC.sh
```

The output is provided in the same folder, including the outC.pdf file with the results image.

Specifically, this synthetic sequence contains the sub-sequences by the following order:

- **R1** - 2k length pseudo-random sequence (seed: 1);
- **R2** - 2k length repetitive sequence (AT-content) from 1 to 2k (seed: 3);
- **R3** - 2k length pseudo-random sequence (seed: 11);
- **R4** - 2k length with copy of R2;
- **R5** - 2k length pseudo-random sequence (seed: 21);
- **R6** - 2k length pseudo-random sequence (seed: 17);
- **R7** - 2k length pseudo-random sequence (seed: 27);
- **R8** - 2k length copy of R6;
- **R9** - 2k length pseudo-random sequence (seed: 31);
- **R10** - 2k length pseudo-random sequence (seed: 47);
- **R11** - 2k length pseudo-random sequence (seed: 37);
- **R12** - 2k length pseudo-random sequence (seed: 55);
- **R13** - 2k length pseudo-random sequence (seed: 67);
- **R14** - 2k length copy of R6;
- **R15** - 2k length pseudo-random sequence (seed: 71);

The following instructions provide support for replicate demonstration C in amino acid sequences

```
./GenSeqCProteins.sh
./DemoCProteins.sh
```

The output is provided in the same folder, including the outCProteins.pdf file with the results image

2.5 Reproducing the “Biological Data results”

These instructions assume that the code has been compiled with the alternative way under the src/ folder. Then, in the main folder of the AlcoR repository, please, type

```
cd herpesvirus/
cp ../src/AlcoR .
chmod +x *.sh
```

In this case, the herpesviruses genomes will already be available in the current folder. Therefore, to compute the low-complexity map, please, type

```
./RunHerpesvirus.sh
```

The output is provided in the same folder as an SVG file with the name mapv.svg. The files with the coordinates will also be available under the same folder.

Then, to reproduce the content of Table S1, please, run

```
./GetPercentageAll.sh
```

2.6 Reproducing the "Large-scale data"

These instructions assume that the code has been compiled with the alternative way under the src/ folder. Reproducibility for two experiments, namely the human and cassava genome low-complexity maps, is provided below.

2.6.1 Human

For preparing this experiment, in the main folder of the AlcoR repository, please, type

```
cd human/
cp ../src/AlcoR .
chmod +x *.sh
```

In this case, for accessing the human complete chromosomes there is the need to download, using the following way

```
./GetHuman.sh
```

After the download, to compute the low-complexity map, please, type

```
./RunHuman.sh
```

The output is provided in the same folder as an SVG file with the name map1.svg. The files with the coordinates will also be available under the same folder.

Then, to reproduce the content of Table S2, please, run

```
./GetPercentageAll.sh
```

2.6.2 Cassava

For preparing this experiment, in the main folder of the AlcoR repository, please, type

```
cd cassava/
cp ../src/AlcoR .
chmod +x *.sh
```

In this case, the cassava diploid complete chromosomes are already available under the same folder but in a compressed way. To decompress, please type

```
./GunzipCassava.sh
```

After the decompression, to compute the low-complexity map, please, type

```
./RunCassava.sh
```

The output is provided in the same folder as an SVG file with the name map2.svg. The files with the coordinates will also be available under the same folder.

To reproduce the content of Table S3, please, run

```
./GetPercentageAll.sh
```

To reproduce the similar regions regarding the end-tip of chromosome 12 (Haplotype B), please, run

```
./RunAdditionalTrace.sh
```

This computation will output the percentage of similarity of each chromosome according to this sub-sequence.

For comparison purposes between AlcoR and RepeatModeler+RepeatMasker, the folder benchmark contains the following scripts that must run

```
./SensitiveCassava.sh
./Comparison.sh
```

The input files must be created or, alternatively, requested to the authors (more than 100 MB of compressed data for each haplotype).

3 Parameters and options of AlcoR

3.1 Main menu

The command to access the main menu with the options/parameters of AlcoR is

```
AlcoR -h
```

This command will output the following content

```
Program: AlcoR [Alignment-free Computation of Low-complexity Regions]
Version: 1.9

Usage: AlcoR <command> [options]

Commands:
      info         Retrieves information of the length and GC
                   percentage of each FASTA read.
      extract      Extracts a sequence of a FASTA file using
                   positional coordinates (independent from
                   the existing headers of the FASTA files).
      mapper       Computes low-complexity regions in a FASTA
                   while providing bidirectional complexity
                   profiles and structural similarity analysis.
      simulation   FASTA sequence simulation with features:
                   file extraction, random generation, sequence
                   modeling (with SNPs specific mutations).
      visual       Computes an SVG file with the respective
                   map containning the low-complexity regions.

Help: AlcoR <command> -h for accessing each command menu.
```

with the sub-commands available (info, extract, mapper, simulation, and visual).

3.2 Info menu

For accessing the sub-menu of extract, the following sub-command must run

```
AlcoR info -h
```

This command will output the following content

```
NAME
      AlcoR info

DESCRIPTION
      It provides length and GC information of each FASTA read.

PARAMETERS

      -h,  --help
           usage guide (help menu),

      -v,  --verbose
           verbose mode (more information),

      -m [INT],  --header-max [INT]
           maximum header size for exporting information,

      [FILE]
           input sequence filename (to analyze) -- MANDATORY,
           FASTA file to retrieve information (last argument).

SYNOPSIS
      AlcoR info [OPTION]... [FILE]

EXAMPLE
      AlcoR info -v -f seq.fa
```

with the options/parameters available.

Accordingly, for each FASTA read, the length (nucleotide or amino acid sequence) and the percentage of G and C bases are registered along with the coordinates of each read. This is a fast and simple implementation that allows extended analysis for comparative purposes.

3.3 Extract menu

For accessing the sub-menu of extract, the following sub-command must run

```
AlcoR extract -h
```

This command will output the following content

```
NAME
      AlcoR extract

DESCRIPTION
```

```
      Extracts a sequence from a FASTA file using coordinates.

PARAMETERS

      -h,  --help
           usage guide (help menu),

      -v,  --verbose
           verbose mode (more information),

      -f,  --fasta
           outputs a FASTA format with the sequence,

      -i [INT],  --init [INT]
           initial position for extracting the sequence,

      -e [INT],  --end [INT]
           end position for extracting the sequence,

      [FILE]
           input sequence filename (to analyze) -- MANDATORY,
           FASTA file for the extraction (last argument).

SYNOPSIS
      AlcoR extract [OPTION]... [FILE]

EXAMPLE
      AlcoR extract -v -f -i 101 -e 301 seq.fa
```

with the options/parameters available.

Notice that the initial and ending position will consider the FASTA headers and this file format's standard line breaks. The output is a FASTA read containing a header with the coordinates and the sequence. Also notice that this feature enables the low-complexity analysis for a specific subsequence of the original one, providing a progressive and flexible analysis.

3.4 Mapper menu

For accessing the sub-menu of mapper, the following sub-command must run

```
AlcoR mapper -h
```

This command will output the following content

```
NAME
      AlcoR mapper

DESCRIPTION
      Computes the low-complexity regions of a sequence (FASTA).

PARAMETERS

      -h,  --help
           usage guide (help menu),

      -v,  --verbose
           verbose mode (more information),

      -n,  --no-size
           does not print the segmented map with the header size,

      -e,  --hide
           it hides (deletes) the final smooth profile,

      -d,  --dna
           considers exclusively DNA alphabet {A,C,G,T},
           it also provides inverted repeats models,
           flag absence considers inversions (without complements),

      -c [INT],  --color [INT]
           Color Hue to be used in visualization,

      -t [FLOAT],  --threshold [FLOAT]
           threshold to segment regions (real),

      -w [INT],  --window [INT]
           window size to smooth the minimum of both directions
```

```
            of compression using a moving average filter (int),

       -i [INT], --ignore [INT]
            ignore lengths of segmented regions below this value,

       -k, --mask
            it masks a FASTA sequence (LCRs appear in lower case),

       -o, --output-mask
            output FASTA filename with the masked sequence,

       -r, --renormalize
            renormalize the positions of a multi-FASTA file,

       -f [STRING], --prefix [STRING]
            use this prefix for the output of renormalize (if on),

       -p, --show-parameters
            show parameters of the models for optimization,

       -s, --show-levels
            show pre-computed compression levels (parameters),

       -l [INT], --level [INT]
            compression level (integer),
            it defines compressibility in balance with computational
            resources (RAM and time), use -s for levels perception,

       [FILE]
            input sequence filename (to analyze) -- MANDATORY,
            FASTA file for the analysis (last argument).

SYNOPSIS
       AlcoR mapper [OPTION]... [FILE]

EXAMPLE
       AlcoR mapper -v -w 10 -m 13:50:0:1:10:0.9/5:10:0.9 -k -o m.fa seq.fa
```

with the options/parameters available. By default, the mapped coordinates are outputted to the stdout (standard output) which can be redirected to a file, namely using

```
Alcor mapper <parameters> seq.fa > stdout.txt
```

The mapper sub-menu has a option to access more information about the compression models, namely through the usage of the following command

```
AlcoR mapper -p
```

This option will output the following model help information

```
-m [C]:[D]:[R]:[I]:[H]:[G]/[S]:[E]:[A]

Template of a target context model.

Parameters:
      [C]: (integer [1;20]) order size of the regular context
           model. Higher values use more RAM but, usually, are
           related to a better compression score.
      [D]: (integer [1;5000]) denominator to build alpha, which
           is a parameter estimator. Alpha is given by 1/[D].
           Higher values are usually used with higher [C],
           and related to confiant bets. When [D] is one,
           the probabilities assume a Laplacian distribution.
      [R]: (integer [0;99999999] memory model. The 0 uses the
           full memory.
      [I]: (integer {0,1,2}) number to define if a sub-program
           which addresses the specific properties of DNA
           sequences (Inverted repeats) is used or not. The
           number 2 turns ON this sub-program without the
           regular context model (only inverted repeats). The
           number 1 turns ON the sub-program using at the same
           time the regular context model. The number 0 does
           not contemple its use (Inverted repeats OFF). The
           use of this sub-program increases the necessary time
           to compress but it does not affect the RAM.
      [H]: (integer [1;254]) size of the cache-hash for deeper
           context models, namely for [C] > 14. When the
           [C] <= 14 use, for example, 1 as a default. The
```

```
            RAM is highly dependent of this value (higher value
            stand for higher RAM).
      [G]: (real [0;1)) real number to define gamma. This value
            represents the decayment forgetting factor of the
            regular context model in definition.
      [S]: (integer [0;20]) maximum number of editions allowed
            to use a substitutional tolerant model with the same
            memory model of the regular context model with
            order size equal to [C]. The value 0 stands for
            turning the tolerant context model off. When the
            model is on, it pauses when the number of editions
            is higher that [C], while it is turned on when
            a complete match of size [C] is seen again. This
            is probabilistic-algorithmic model very usefull to
            handle the high substitutional nature of genomic
            sequences. When [S] > 0, the compressor used more
            processing time, but uses the same RAM and, usually,
            achieves a substantial higher compression ratio. The
            impact of this model is usually only noticed for
            [C] >= 14.
      [E]: (integer [1;5000]) denominator to build alpha for
            substitutional tolerant context model. It is
            analogous to [D], however to be only used in the
            probabilistic model for computing the statistics of
            the substitutional tolerant context model.
      [A]: (real [0;1)) real number to define gamma. This value
            represents the decayment forgetting factor of the
            substitutional tolerant context model in definition.
            Its definition and use is analogus to [G].
```

Nevertheless, several compression level models are already pre-computed. For accessing these pre-levels, the following command must run

```
AlcoR mapper -s
```

This option will output the following fifteen levels and model setups

```
Level  1: -m 3:10:500:0:1:0.97/0:0:0
Level  2: -m 3:10:500:2:1:0.97/0:0:0
Level  3: -m 3:10:500:1:1:0.97/0:0:0
Level  4: -m 5:20:500:0:1:0.97/0:0:0
Level  5: -m 5:20:500:2:1:0.97/0:0:0
Level  6: -m 5:20:500:1:1:0.97/0:0:0
Level  7: -m 8:20:800:0:0:0.9/0:0:0
Level  8: -m 8:20:800:1:0:0.9/0:0:0
Level  9: -m 13:50:0:1:10:0.9/5:10:0.9
Level 10: -m 12:50:1000:0:0:0.9/0:0:0
Level 11: -m 12:50:1000:1:0:0.9/0:0:0
Level 12: -m 12:50:1000:2:0:0.9/0:0:0
Level 13: -m 6:20:750:0:0:0.9/0:0:0 -m 13:20:1500:0:0:0.9/2:10:0.9
Level 14: -m 6:20:750:1:0:0.9/0:0:0 -m 13:20:1500:1:0:0.9/2:10:0.9
Level 15: -m 6:20:750:2:0:0.9/0:0:0 -m 13:20:1500:2:0:0.9/2:10:0.9
```

The default level is 9.

3.5 Simulation menu

For accessing the sub-menu of simulation, the following sub-command must run

```
AlcoR simulation -h
```

This command will output the following content

```
NAME
      AlcoR simulation

DESCRIPTION
      Simulation of FASTA sequences with specific features.

PARAMETERS

      -h,  --help
           usage guide (help menu),

      -v,  --verbose
           verbose mode (more information),

      -n,  --no-dna
```

```
            Does not consider a DNA alphabet {A,C,G,T},
            it provides possible inversions without complements,

      -a [STRING], --alphabet [STRING]
            alphabet to consider (Default: ACGT),

      -fs [FEATURES], --file-segment [FEATURES]
            FASTA file segment features:
            |     [init:end:ir:seed:subs:adds:dels:file]
            |     ...
            init [INT]   - initial position of the segment,
            end  [INT]   - ending position of the segment,
            ir   [INT]   - segment inverted if 1, otherwise 0,
            seed [INT]   - initial number for random generation,
            subs [FLOAT] - probability of substitution mutation,
            adds [FLOAT] - probability of addition mutation,
            dels [FLOAT] - probability of deletion mutation,
            file [FILE]  - FASTA filename for extracting segment,

      -rs [FEATURES], --rand-segment [FEATURES]
            Random segment features:
            |     [size:ir:seed:subs:adds:dels]
            |     ...
            size [INT]   - length of the segment,
            ir   [INT]   - segment inverted if 1, otherwise 0,
            seed [INT]   - initial number for random generation,
            subs [FLOAT] - probability of substitution mutation,
            adds [FLOAT] - probability of addition mutation,
            dels [FLOAT] - probability of deletion mutation,

      -ms [FEATURES], --model-segment [FEATURES]
            Model segment features:
            |     [size:ctx:bet:ir:seed:subs:adds:dels:file]
            |     ...
            size [INT]   - length of the segment,
            ctx  [INT]   - context to model and simulate,
            bet  [INT]   - intensity for higher bet strength,
            ir   [INT]   - segment inverted if 1, otherwise 0,
            seed [INT]   - initial number for random generation,
            subs [FLOAT] - probability of substitution mutation,
            adds [FLOAT] - probability of addition mutation,
            dels [FLOAT] - probability of deletion mutation,
            file [FILE]  - FASTA filename for learning model,

SYNOPSIS
      AlcoR simulation [OPTION]... > output.fa

EXAMPLE
      AlcoR simulation -rs 50:0:1:0.1:0:0 -ms 80:7:50:0:7:0:0:0:x.fa
```

with the options/parameters available.

3.6 Visual menu

For accessing the sub-menu of visual, the following sub-command must run

```
AlcoR visual -h
```

This command will output the following content

```
NAME
      AlcoR visual

DESCRIPTION
      Creates an SVG map with the identified regions.

PARAMETERS

      -h, --help
            usage guide (help menu),

      -v, --verbose
            verbose mode (more information),

      -w [INT], --width [INT]
            horizontal width (thickness) of each bar,

```

```
      -s [INT],  --space [INT]
           space between each bar,

      -e [INT],  --enlarge [INT]
           enlargement of each region (increase visibility),

      -c,  --strict-corner
           it paints each bar with strict corners (NO round),

      -b [STRING],  --back-color [STRING]
           background color in RGB format (example: FFFFFF),

      -a [STRING],  --border-color [STRING]
           bar border color in RGB format (example: 000000),

      -o [FILE],  --output [FILE]
           filename of the SVG output map,

      [FILE]:[FILE]:...
           input position filenames (to analyze) -- MANDATORY,
           multiple files can be used with : split token.

SYNOPSIS
      AlcoR visual [OPTION]... [FILE]:...

EXAMPLE
      AlcoR visual -v -o map.svg pos1.txt:pos2.txt:pos3.txt
```

with the options/parameters available.

The input files containing the coordinates of the mapped regions can be set separately as input through the ‘:’ symbol splitter. This option allows loading multiple maps in a single SVG image, providing the ideal conditions for sequence comparison through the ideogram style, for example, multiple chromosomal maps from a plant genome or multiple similar viruses comparison.

References

[1] Björn Grüning, Ryan Dale, Andreas Sjödin, Brad A Chapman, Jillian Rowe, Christopher H Tomkins-Tinch, Renan Valieris, and Johannes Köster. Bioconda: sustainable and comprehensive software distribution for the life sciences. *Nature methods*, 15(7):475–476, 2018.
