# Supplementary material for: AlcoR: alignment-free simulation, mapping, and visualization of low-complexity regions in biological data
Source: Gigascience. 2023 Dec 13;12:giad101. doi: 10.1093/gigascience/giad101 (PMC10716826; doi:10.1093/gigascience/giad101)
Supplement: giad101_Response_to_Reviewer_Comments_Original_Submission [file giad101_response_to_reviewer_comments_original_submission.pdf]

Author's Response To Reviewer Comments

(PDF version attached)

Dear Editor,

We greatly appreciate the opportunity given to revise our manuscript. We would like to thank the Review-
ers, whose suggestions allowed us to improve our manuscript in many ways. We have now addressed the points raised by them, as outlined in blue in this revision letter. Following their suggestions, appropriate changes have been introduced to the manuscript, as shown also in blue. We also reinforce the complexity in benchmarking these tools, counterintuitively given by the absence of a true gold standard (Kolmogorov complexity) and the different between these tools. We hope we have been able to address their concerns and
that our manuscript is now suitable for publication at GigaScience.

Reviewer 1

Reviewer: The authors present a new method for for reference-free and alignment-free method for the low-complexity region analysis (LCRs) in FASTA data. The method is well demonstrated on the complete human genome and the haplotype-resolved chromosome pairs of a heterozygous diploid African cassava cultivar. The tool is well documented and an open-source without any dependencies on external software. For better presentation, a benchmarking table against competing methods would be nice for readers.

Authors: We thank the Reviewer for the sugestions and comments. Measuring the complexity of sequences is approximating the Kolmogorov complexity [1] and, since it is an uncomputable measure, we can only provide an upper bound approximation. Therefore, regions detected by LCR mappers are low-complexity regions (if mappers do not underestimate) but regions that are not mapped may be of low-complexity since
the models may not be able to identify it. Accordingly, there is no computable gold standard for benchmark-
ing LCR detection because the exact reference values are not known. We could argue that simulations may provide a true gold standard for benchmark but the reality is that simulations based on linear functions create
sequences that are all of low complexity because there is a simple program to explain them. Nevertheless, they can be used as indicative only, since mapper models usually do not contain the ability to identify the origin from simulations. We have reinforced in the manuscript this explanation, strengthening that putting this data into a table form may not be appropriate.

Reviewer 2

Reviewer: The authors have presented a means to rapidly extract LCR from fasta files from various organ-
isms. The tool covers compression of the fasta files, simulation of fasta data with various parameters, and the identification of LCR with various characteristics. While this work clearly represents substantial work 1in developing the compression method and means to identify LCRs under different conditions it seems that
there may be some useful tools/options that are missing.

Authors: We thank the Reviewer for the suggestions and appreciate the comments.

Reviewer: It’s not clear to me if this is a new compression method for fasta? or an existing strategy. If new how does it compare to other compression strategies? The main point of this work isn’t to create new or better compression, but it would be useful to know how it compares.

Authors: The AlcoR compression scheme is based on GeCo3, an existing strategy [2]. GeCo3 is a state-
of-the-art data compressor for genomic sequences. Besides the GeCo3 modeling scheme, AlcoR also uses

a new type of model that incorporates locality estimation capabilities. This is a very important feature to split local from distant LCRs. Therefore, if we would benchmark AlcoR against GeCo3 we would get very similar results, where perhaps AlcoR would have slightly higher compression gains given the additional use of these new local models. However, AlcoR is not intended to compress data for storage purposes but only for analysis. For this reason, AlcoR does not include an arithmetic encoder that is fundamental for the final part of compressing the data for storage purposes. This is not needed because, for analysis purposes, AlcoR
requires only estimating the probabilities that the mixing of the models provides. In practice, for speed purposes, we calculate the number of bits provided by the arithmetic encoder with identical accuracy. This number of bits is very close to the arithmetic encoder, for example, in gigabytes of data it only differs a few
kilobytes. Without the arithmetic encoder module, AlcoR can not be considered a “true” data compressor but rather a compression-based method for analysis purposes. Moreover, AlcoR uses parallel bidirectional complexity profiles, which is equivalent to compressing the sequence using both ways, namely from the beginning to the end and from the end to the beginning. For a data compression tool for storage purposes, this double compression does not make sense because we would be doubling the number of bits needed to represent the sequence. However, for AlcoR is fundamental to map the LCRs associated with distant patterns.

Reviewer: How was the comparison of RepeatMasker results to AlcoR carried out? I can’t tell if there is an option to output a Bed file for comparative studies. I am guessing the SVG file was loaded to NCBIv then extracted as a Bed file? This is a feature that would be important for analysis - particularly across many
genomes. Visual inspection will not take the place quantitative analysis - for example a pan genome analysis
of TE insertion sites across hundreds of sorghum varieties. You mention coordinate retrieval in discussion but I do not see where that is implemented.

Authors: The comparison of RepeatMasker with AlcoR has been carried out through the intersection of the LCRs mapped. This procedure is computed using script Comparison.sh while the associated description is available in Supplementary Subsection 2.6.2. Notice that this is only indicative as we explained in the text and to Reviewer 1. Accordingly, AlcoR outputs the positions of each segmented region, namely the initial and ending coordinate. By default, these coordinates are outputted to the stdout (we now reinforce this information at the supplementary material). Therefore, this feature already exists. A tiny indicative example
of such coordinates output is the following:
1 501 100
3057 3584 100
5610 6683 100
8802 8922 100
Another example can be seen at the website pipeline, specifically at the first pipeline description (https://cobilab.github.io/alcor/#Pipelines) where the coordinates are redirected to the LCRs.csv file.

Reviewer: What about quality scores? The simulation includes means by which to test if different mutation rates can be tolerated while still identifying LCRs, which is useful for high error data. But wouldn’t using quality scores also help in accurately predicting LCRs? I would speculate that high error data would make it harder to predict LCRs that have ”real” mutations. The quality scores could be used as a threshold to decide
if the mutation is ”real” or not.

Authors: Quality scores are contained in FASTQ (not FASTA) files which derive directly from sequencing outputs. LCRs are usually mapped in FASTA file formats, which are generated after reconstructing (or assembly) the genomes from FASTQ inputs. For example, RepeatMasker and AlcoR use FASTA formats. Therefore, in FASTA formats quality scores are not present and, hence, they can not be used to improve the
prediction of LCRs. Nevertheless, in the reconstruction process, it is possible to apply trimming and filtering methodologies to discard or improve the quality of this data. On the other hand, this idea is an interesting

methodology but will require to incorporate reconstruction at its core for the prediction of LCRs. Thank you for the hint about a potentially better methodology that can work at the FASTQ level.

Reviewer: Can you output the level of deviation from an expected LCR? In the discussion you suggest viral integration as a use case. Would one be able to extract the level of deviation from the canonical viral sequence?

Authors: We are not sure what the Reviewer means with the level of deviation in this case. AlcoR, besides the availability of the LCR coordinates output, masked sequence, and visual map, provides the filtered and smooth profile automatically for a file. This file contains the level of complexity associated with each symbol
of the sequence to map. Therefore, this can be seen as a scoring number that by the average from the initial
and end coordinates quantifies how complex is a certain region.

References
[1] A. N. Kolmogorov. Three approaches to the quantitative definition of information. Problems of information transmission, 1965.
[2] M. Silva, D. Pratas, A. J. Pinho. Efficient DNA sequence compression with neural networks. GigaScience, 2020.
