# Supplementary material for: AlcoR: alignment-free simulation, mapping, and visualization of low-complexity regions in biological data
Source: Gigascience. 2023 Dec 13;12:giad101. doi: 10.1093/gigascience/giad101 (PMC10716826; doi:10.1093/gigascience/giad101)
Supplement: giad101_Response_to_Reviewer_Comments_Revision_1 [file giad101_response_to_reviewer_comments_revision_1.pdf]

Author's Response To Reviewer Comments

Thank you!
Here are the changes request.
Kind regards.
