# Supplementary material for: AlcoR: alignment-free simulation, mapping, and visualization of low-complexity regions in biological data
Source: Gigascience. 2023 Dec 13;12:giad101. doi: 10.1093/gigascience/giad101 (PMC10716826; doi:10.1093/gigascience/giad101)
Supplement: giad101_Reviewer_2_Report_Original_Submission — Sara Goodwin -- 9/5/2023 Reviewed [file giad101_reviewer_2_report_original_submission.pdf]

Reviewer Report

Title: AlcoR: alignment-free simulation, mapping, and visualization of low-complexity regions in biological data

Version: Original Submission **Date:** 9/5/2023

Reviewer name: Sara Goodwin

Reviewer Comments to Author:

The authors have presented a means to rapidly extract LCR from fasta files from various organisms. The tool covers compression of the fasta files, simulation of fasta data with various parameters, and the identification of LCR with various characteristics. While this work clearly represents substantial work in developing the compression method and means to identify LCRs under different conditions it seems that there may be some useful tools/options that are missing.It's not clear to me if this is a new compression method for fasta? or an existing strategy. If new how does it compare to other compression strategies? The main point of this work isn't to create new or better compression, but it would be useful to know how it compares.How was the comparison of RepeatMasker results to AlcoR carried out? I can't tell if there is an option to output a Bed file for comparative studies. I am guessing the SVG file was loaded to NCBIv then extracted as a Bed file? This is a feature that would be important for analysis - particularly across many genomes. Visual inspection will not take the place quantitative analysis - for example a pan genome analysis of TE insertion sites across hundreds of sorghum varieties. You mention coordinate retrieval in discussion but I do not see where that is implemented.What about quality scores? The simulation includes means by which to test if different mutation rates can be tolerated while still identifying LCRs, which is useful for high error data. But wouldn't using quality scores also help in accurately predicting LCRs? I would speculate that high error data would make it harder to predict LCRs that have "real" mutations. The quality scores could be used as a threshold to decide if the mutation is "real" or not.Can you output the level of deviation from an expected LCR? In the discussion you suggest viral integration as a use case. Would one be able to extract the level of deviation from the canonical viral sequence?

Level of Interest

Please indicate how interesting you found the manuscript: Choose an item.

Quality of Written English

Please indicate the quality of language in the manuscript: Choose an item.

Declaration of Competing Interests

Please complete a declaration of competing interests, considering the following questions:

- Have you in the past five years received reimbursements, fees, funding, or salary from an organisation that may in any way gain or lose financially from the publication of this manuscript, either now or in the future?
- Do you hold any stocks or shares in an organisation that may in any way gain or lose financially from the publication of this manuscript, either now or in the future?
- Do you hold or are you currently applying for any patents relating to the content of the manuscript?
- Have you received reimbursements, fees, funding, or salary from an organization that holds or has applied for patents relating to the content of the manuscript?
- Do you have any other financial competing interests?
- Do you have any non-financial competing interests in relation to this paper?

If you can answer no to all of the above, write 'I declare that I have no competing interests' below. If your reply is yes to any, please give details below.

I declare that I have no competing interests

I agree to the open peer review policy of the journal. I understand that my name will be included on my report to the authors and, if the manuscript is accepted for publication, my named report including any attachments I upload will be posted on the website along with the authors' responses. I agree for my report to be made available under an Open Access Creative Commons CC-BY license (http://creativecommons.org/licenses/by/4.0/). I understand that any comments which I do not wish to be included in my named report can be included as confidential comments to the editors, which will not be published.

Choose an item.

To further support our reviewers, we have joined with Publons, where you can gain additional credit to further highlight your hard work (see: https://publons.com/journal/530/gigascience). On publication of this paper, your review will be automatically added to Publons, you can then choose whether or not to claim your Publons credit. I understand this statement.

Yes Choose an item.
